# *qDTY*_*12*.*1*_: a locus with a consistent effect on grain yield under drought in rice

**DOI:** 10.1186/1471-2156-14-12

**Published:** 2013-02-26

**Authors:** Krishna Kumar Mishra, Prashant Vikram, Ram Baran Yadaw, BP Mallikarjuna Swamy, Shalabh Dixit, Ma Teresa Sta Cruz, Paul Maturan, Shailesh Marker, Arvind Kumar

**Affiliations:** 1 International Rice Research Institute (IRRI), DAPO Box 7777, Metro Manila, Philippines; 2 Sam Higginbottom Institute of Agriculture, Technology & Sciences, Allahabad 211007UP, India; 3 Nepal Agricultural Research Council (NARC), Kathmandu, Nepal

**Keywords:** Drought, Grain yield, Rice, QTL

## Abstract

**Background:**

Selection for grain yield under drought is an efficient criterion for improving the drought tolerance of rice. Recently, some drought-tolerant rice varieties have been developed using this selection criterion and successfully released for cultivation in drought-prone target environments. The process can be made more efficient and rapid through marker-assisted breeding, a well-known fast-track approach in crop improvement. QTLs have been identified for grain yield under drought with large effects against drought-susceptible varieties. Most of the identified QTLs show large QTL × environment or QTL × genetic background interactions. The development of mapping populations in the background of popular high-yielding varieties, screening across environments, including the target environments, and the identification of QTLs with a consistent effect across environments can be a suitable alternative marker-assisted breeding strategy. An IR74371-46-1-1 × Sabitri backcross inbred line population was screened for reproductive-stage drought stress at the International Rice Research Institute, Philippines, and Regional Agricultural Research Station, Nepalgunj, Nepal, in the dry and wet seasons of 2011, respectively. A bulk segregant analysis approach was used to identify markers associated with high grain yield under drought.

**Results:**

A QTL, *qDTY*_*12*.*1*_, significantly associated with grain yield under reproductive-stage drought stress was identified on chromosome 12 with a consistent effect in two environments: IRRI, Philippines, and RARS, Nepalgunj, Nepal. This QTL explained phenotypic variance of 23.8% and contributed an additive effect of 45.3% for grain yield under drought. The positive QTL allele for *qDTY*_*12*.*1*_ was contributed by tolerant parent IR74371-46-1-1.

**Conclusions:**

In this study, *qDTY*_*12*.*1*_ showed a consistent effect across environments for high grain yield under lowland reproductive-stage drought stress in the background of popular high-yielding but drought-susceptible recipient variety Sabitri. *qDTY*_*12*.*1*_ was also reported previously [*Crop Sci* 47:507–516, 2007] to increase grain yield under upland reproductive-stage drought stress situations. *qDTY*_*12*.*1*_ is the only QTL reported so far in rice to have shown a large effect against multiple recipient genetic backgrounds as well as under highly diverse upland and lowland rice ecosystems. *qDTY*_*12*.*1*_ can be successfully introgressed to improve grain yield under drought of popular high-yielding but drought-susceptible lowland as well as upland adapted varieties following marker-assisted breeding.

## Background

Drought is one of the most important abiotic stresses hampering rice productivity in rainfed areas. In Asia more than 23 million ha of rice-growing area are rainfed [1]. Eastern India and adjoining areas of Nepal occupy a large drought-affected area with an estimate of around 17 million ha [2]. The green revolution had little impact in rainfed ecosystems [3]. Farmers in these areas are growing popular varieties originally bred for irrigated ecosystems [4]. The slow progress in developing rice varieties for drought-prone areas is mainly due to the complex nature of drought-tolerance mechanisms; large genotype × environment, QTL × environment and QTL × recipient genetic background interactions; and the absence of QTLs with a large and consistent effect against high-yielding but drought-susceptible varieties. Not only the interactions but also the complex nature of drought, which affects the rice plant at all stages of crop growth, and its relation with a number of physiological mechanisms and biochemical pathways further complicate the problem. Therefore, a strategy of screening in different environments, particularly in the target population of environments (TPE), is advocated for developing varieties with broader adaptation [5]. Trait selection is another important concern in drought-tolerance rice breeding programs. Recent studies have reported grain yield (GY) under drought as an effective selection criterion for the development of drought-tolerant rice varieties [6,7]. During the last few years, several varieties have been developed and released in India, Nepal and Bangladesh following GY under reproductive-stage drought stress (RS) as a selection criterion [8].

The ultimate aim of a plant breeder is to identify rice genotypes with a stable performance across a range of environments, which is a time-consuming process. A marker-assisted breeding (MAB) strategy, advocated to be a fast-track approach in rice improvement for drought-prone environments, can be a suitable alternative strategy as a solution [4,9]. The marker-assisted backcrossing (MABC) approach has been used to improve the drought tolerance of high-yielding, popular, farmer-adapted varieties grown on a large scale [10]. Several major quantitative trait loci (QTLs) have been identified for high GY under RS [4,9,11]. A large-effect QTL, *qDTY*_*12*.*1*_, was identified for GY under RS in upland situations in a Vandana/Way Rarem population explaining 51% of the genetic variance [9]. Two other QTLs, *qDTY*_*1*.*1*_ and *qDTY*_*3*.*1*_, were identified for lowland RS. *qDTY*_*1*.*1*_ showed a consistent effect in three different genetic backgrounds, Swarna, IR64 and MTU1010, explaining phenotypic variance up to 16.9% [4]. *qDTY*_*3*.*1*_, identified in an Apo/Swarna population, explained 31% of the genetic variance in lowland RS [11].

QTLs with large and consistent effects are worthy for use in marker-assisted selection (MAS) to improve the drought tolerance of presently cultivated varieties [12]. The most suitable QTL for drought would be one that can overcome QTL × genetic background, QTL × environment and QTL × ecosystem effects. To identify genomic regions with a consistent effect across environments, large mapping populations need to be screened in different environments. Genotyping and phenotyping of large mapping populations involve high cost and much effort. The genotyping cost can be reduced through using a bulk segregant analysis (BSA) approach. BSA has been suggested as a cost-effective and powerful genotyping method in the identification of QTLs for high GY under RS [4,11,13]. BSA involves pooling of DNA of the phenotypic extremes and genotyping along with the parents to identify markers linked with the trait of interest [14].

The identification and introgression of QTLs in the background of elite rice varieties could be helpful in MAB [4]. Sabitri is a popular variety of the lowland rice ecosystems of Nepal and adjoining parts of India but it is highly susceptible to drought. Our study was undertaken with the objective to identify QTLs with a large and consistent effect for GY under RS in the background of popular recipient rice variety Sabitri.

## Methods

The study was conducted at the International Rice Research Institute (IRRI), Los Baños, Laguna, Philippines, and at the Regional Agricultural Research Station (RARS), Nepalgunj, Nepal, in DS2011 (dry season, January-May) and WS2011 (wet season, June-November), respectively. The dry-season experiment was sown on December 17, 2011, and the wet-season experiment was seeded on July 22, 2011.

### Plant materials

A BC_1_F_3:5_ backcross inbred line (BIL) population developed from the cross of ‘IR74371-46-1-1’ and ‘Sabitri’ was used for this study. IR74371-46-1-1 is a drought-tolerant variety released in Nepal as ‘Sookha Dhan-1’ [8]. IR74371-46-1-1 is a backcross-derived line from Way Rarem (Way Rarem/2*IR55419-04). Way Rarem is an upland adapted variety of Indonesia. Way Rarem is known to contribute a large-effect QTL for high grain yield under drought [9]. In contrast to IR74371-46-1-1, Sabitri is highly susceptible to drought. The BIL population used in the study was developed by crossing IR74371-46-1-1 with Sabitri twice and BC_1_F_1_ seeds were selfed. One BC_1_F_3_ seed from each BC_1_F_2_ plant was selected and bulked to make BC_1_F_3_ seeds. BC_1_F_3_ seeds were grown and each plant was harvested individually. The BC_1_F_3:4_ plants were grown and harvested in bulk. The BC_1_F_3:5_ lines were screened for GY under RS at IRRI in DS2011 and in Nepalgunj, Nepal, in WS2011.

### Phenotyping

The IR74371-46-1-1/2* Sabitri BIL population was screened for GY under RS in DS2011 at IRRI and in WS2011 at Nepalgunj, Nepal. Screening under non-stress (NS) situations was carried out in WS2011 at Nepalgunj, Nepal. A total of 294 BIL lines were screened at IRRI in DS2011 under severe lowland drought conditions while 234 lines were screened in Nepal under lowland drought stress and non- stress conditions. Experiments were laid out in an alpha lattice design in two replications with a 5-meter (m) single-row plot having row spacing of 0.2 m. Twenty-one-day-old seedlings were transplanted. A single seedling per hill was transplanted with 0.2-m spacing between the hills in the row. Throughout the crop season, there were 5 cm of standing water in the NS experiment and the fields were drained before harvesting. Nitrogen, phosphorus and potassium (NPK) were applied at 120:30:30 kg ha^-1^. In order to control snails, Bayluscide (niclosamide, 0.25 kg a.i. ha^-1^) was sprayed just after transplanting. At 4 days after transplanting (DAT), Sofit (pretilachlor ± safener, 0.3 kg a.i. ha^-1^) was sprayed to control weeds, followed by Furadan (carbofuran, 1 kg a.i. ha^-1^) at 5 DAT and Cymbush (cypermethrin, 1 L ha^-1^) ± Dimotrin (cartap hydrochloride, 0.25 kg a.i. ha^-1^) at 16 DAT to control insect pests. Fields were drained at 30 DAT and irrigation was withheld to impose drought stress at the reproductive stage. Stress was continued until severe leaf rolling (LR) was observed in at least 75% of the population lines and water table depth remained below 100 cm for more than 2 weeks. Life-saving irrigation was provided thereafter through flash flooding and water was drained after 24 hours to impose a second cycle of drought stress [7]. Severe LR was not observed again after providing the life-saving irrigation in both DS2011RS and WS2011RS experiments. The second cycle of stress continued up to maturity. Water table depth was measured by inserting a 1.1-meter PVC (polyvinyl carbonate) pipe in experimental fields at regular intervals. Pipes were inserted to 1.0-meter depth and 10 centimeters of pipe remained above the soil surface. Depletion in the water table was measured through a meter scale daily after the onset of the stress. Observations were recorded for days to 50% flowering (DTF), plant height (PHT), biomass (BIO), GY, harvest index (HI), LR and panicle number (PAN). DTF was recorded when 50% of the panicles of the plants of each plot were exserted. PHT (cm) was measured at maturity from the soil surface to the tip of the panicle on the main tiller from three random plants of each plot and then the mean was calculated. BIO (g m^-2^ converted to kg ha^-1^) was taken from a 1-m linear length in each plot and then oven-dried. Samples were then weighed and threshed for grain weight. HI was estimated with the formula:

$$\mathrm{HI}=\frac{\mathrm{GY}}{\mathrm{BIO}}$$

where HI is harvest index, GY is grain yield and BIO is total biomass. Harvesting for GY (g m^-2^ converted to kg ha^-1^) was done at physiological maturity. Samples were harvested and dried to 12% moisture before weighing [11].

### Genotyping

Leaves were collected from each plot from the first replicate of the stress experiment in the DS2011 experiment at IRRI. One leaf from an alternate plant of each plot was taken and bulked so that the bulk represented one BC_1_F_3:5_ line. Samples were freeze-dried, cut and placed in eppendorf tubes and ground using a GENO grinder. DNA extraction was carried out by the modified CTAB method and DNA was stored in the deep-well plates (Axygen Scientific, California, USA) [15]. Quantification of the DNA samples was carried out on 0.8% agarose gel. Concentration of the DNA samples was adjusted to ~25 ng μL^-1^. A reaction mixture of 15 μL including 50 ng DNA, 1X PCR buffer, 100 μM dNTPs, 250 μM primers and 1 unit *Taq* polymerase enzyme was used for PCR amplification. PCR products were resolved on 8% non-denaturing polyacrylamide gels using a mini-vertical electrophoresis system (CBS Scientific, model MGV-202-33) [16]. A parental polymorphism survey was carried out between IR74371-46-1-1 and Sabitri with 682 rice simple sequence repeat (SSR) markers (ResGen, Invitrogen Corporation, Huntsville) from already available rice genetic and sequence maps [17-19].

BSA was carried out to identify the QTL for GY under RS using 10% of the tail lines. DNA of 5% of the lines with the highest GY and 5% with the lowest GY under RS was extracted and pooled separately to make two bulks: bulk high and bulk low [20]. The concentration of all DNA samples was equalized before pooling. Four DNA samples, including two bulks (bulk high and bulk low) and two parents (IR74371-46-1-1 and Sabitri), were genotyped with 106 polymorphic SSR markers [4]. The significant marker identified in BSA, RM28166, was run on the whole population and single-marker analysis was done. Thereafter, five additional markers (RM28048, RM28089, RM28099, RM511 and RM28199) were run on a whole population to determine the confidence interval of the QTL region. A similar procedure was followed by earlier workers in identifying large-effect drought GY QTLs via BSA [4,13,20].

### Statistical analysis

Statistical analysis was carried out using CROPSTAT software version 7.2.3. The linear mixed model was used for analysis of variance (ANOVA). Entry means were estimated within the season using a model in which replications and blocks within replicates were random and entries remained fixed. To estimate the combined mean of RS experiments conducted at IRRI, Philippines, and RARS, Nepal, location effects were also taken as random. Variance components were estimated to calculate the broad-sense heritability by keeping all the sources of variation as random. Heritability (*H*) was calculated using the formula:

$$H=\frac{V_{g}}{V_{g}+V_{e}/r}$$

where V_g_ is genotypic variance, V_e_ is error variance and r is the number of replications.

### QTL analysis

QTL analysis for *qDTY*_*12*.*1*_ was conducted with six markers, including marker RM28166 identified in BSA and five markers adjacent to it. Details of primers are provided in Additional file 1. QTL analysis was carried out with the entry means of phenotypic traits for stress trials in both seasons as well as with the combined mean across two seasons of stress experiments. QTL analysis was also conducted for the NS experiment. QTL analysis was performed through QTL network v.2.1 [21]. Mixed model–based composite interval mapping was performed through 1000 permutation tests to calculate the critical *F*-value and to control the genome-wide type I error. Phenotypic variance was estimated through QGene v4.3.10 software [22].The significant marker intervals were detected and additive effect was calculated using the formula:

$$\mathrm{AE}\%=\frac{\mathrm{AE}\times 100}{\mathrm{PM}}$$

where AE is the additive effect and AE% is additive effect as a percentage of the population means designated as PM. One million bases on a rice chromosome were assumed to be equivalent to approximately 3.92 cM while determining the genetic distances [19].

### *qDTY*_*12*.*1*_ allele analysis

*qDTY*_*12*.*1*_ identified in this study in the IR74371-46-1-1 × Sabitri population was previously identified in a Vandana × Way Rarem population [9]. To better understand the allele contribution for *qDTY*_*12*.*1*_, an allele survey was carried out using *qDTY*_*12*.*1*_ markers RM28089, RM511, RM28166 and RM28199 among four parents, Vandana, Way Rarem, Sabitri and IR74371-46-1-1.

## Results

### Phenotypic variances in the population

In DS2011, during the flowering period, the water table was below −80 KPa except for one day when it reached −60 KPa (Additional file 2) because of the three rainy days, March 4–6 (rainfall of 9.6 mm). In WS2011, there was no rain during the stress period and the water table depth was around −100 cm throughout the flowering period (Additional file 3). Phenotypic variations in genotypes were observed for all the traits recorded under RS and NS experiments. Trial means, range and broad-sense heritability of the traits measured in RS and NS carried out at IRRI, Philippines (DS2011RS), as well as RARS, Nepal (WS2011RS and WS2011NS), are presented in Table 1. The NS experiment was carried out in Nepal in the wet season (WS2011) and no NS experiment was carried out at IRRI in the dry season. GY under RS ranged from 0 to 4320 kg ha^-1^ with a mean GY of 707 kg ha^-1^ in DS2011 and 885 kg ha^-1^ in the WS2011 stress trial, whereas mean GY under NS conditions was 4639 kg ha^-1^, with a range from 1500 to 7500 kg ha^-1^. Heritability of GY was 0.81 and 0.71 for RS in DS2011 and WS2011, respectively, and 0.86 for NS in WS2011 (Table 1). DTF of the population lines ranged from 76 to 112 days, with a mean of 95 and 88 days in DS2011 and WS2011 RS experiments. Under NS, DTF ranged from 78 to 95 days, with a mean of 87 days. Heritability of DTF ranged from 0.91 to 0.97. PHT ranged from 53 to 118 cm, with a trial mean of 78 and 94 cm in DS2011 and WS2011 Rs experiments. Under NS conditions, PHT ranged from 68 to 138 cm, with a trial mean of 111 cm. Heritability of PHT ranged from 0.67 to 0.72. LR, BIO, HI and PAN were recorded in RS and NS experiments of WS2011 only. LR ranged from 1 to 7, with a trial mean score of approx. 5. Heritability of LR was 0.89. BIO ranged from 1200 to 9048 kg ha^-1^, with a trial mean of 3882 kg ha^-1^ under RS, whereas, under NS, it was 4500–21380, with a trial mean of 12090 kg ha^-1^. The heritability estimates for BIO under RS and NS experiments were 0.77 and 0.84, respectively. HI ranged from 0.01 to 0.62, with a trial mean of 0.20 under RS, whereas, under NS, the range was 0.20-0.70, with a trial mean of 0.30. Heritability for HI under RS and NS was 0.47 and 0.79, respectively (Table 1).

**Table 1:** Trial means, range, standard deviation (SD) and broad-sense heritability (H) for grain yield and yield components under RS and NS conditions of IRRI (DS2011) and Nepal (WS2011RS and WS2011NS)

**Trait**	**Season**	**Trial mean**	**IR74371-46-1-1**	**Sabitri**	**Range**	**SD**	**H**
GY (kg ha^-1^)	DS2011RS	707	3000	0	0-3499	994	0.81
	WS2011RS	885	3411	69	15-4320	877	0.71
	WS2011NS	4639	5308	4625	1500-7500	1137	0.86
DTF (days)	DS2011RS	95	85	NF	79-112	5	0.91
	WS2011RS	88	86	104	76-105	5	0.97
	WS2011NS	87	85	104	78-95	3	0.94
PHT (cm)	DS2011RS	78	98	98	53-104	10	0.67
	WS2011RS	94	96	94	65-118	12	0.72
	WS2011NS	111	117	105	68-138	13	0.68
LR	WS2011RS	5	1	7	1-7	2	0.89
	WS2011NS	-	-	-	-	-	-
BIO	WS2011RS	3882	7564	3884	1200-9048	1337	0.77
	WS2011NS	12090	13308	12319	4500-21380	2424	0.84
HI	WS2011RS	0.2	0.4	0.1	0.01-0.62	0.2	0.47
	WS2011NS	0.3	0.3	0.3	0.20-0.70	0.1	0.79
Pan	WS2011RS	7	13	7	4-14	2	0.94
	WS2011NS	11	12	11	7-19	2	0.86

*DS2011RS*: RS experiment of dry season at IRRI; *WS2011RS*: RS experiment of wet season in Nepal; *WS2011NS*: NS experiment of wet season in Nepal; *GY*: Grain yield; *DTF*: Days to 50% flowering; *PHT*: Plant height; *LR*: Leaf rolling; *BIO*: Biomass; *HI*: Harvest index; *PAN*: Panicle number; *NF*: Did not flower; LR, BIO, HI and PAN were measured in WS2011RS only.

### Phenotypic correlations

Phenotypic correlations between GY and other traits were calculated and are presented in Table 2. GY was negatively correlated with DTF in DS2011 and WS2011 RS experiments. This correlation was positive in the NS experiment. A positive but significant correlation was observed between GY and PHT in the NS experiment; however, it was non-significant in RS experiments. A strong positive correlation was observed between GY and BIO and HI in RS experiments.

**Table 2:** Correlation of GY with other traits under RS and NS conditions

**Ecosystem/season**	**DTF**	**PHT**	**LR**	**BIO**	**HI**
WS2011NS	0.23*	0.27*	NR	0.35*	0.64*
WS2011RS	−0.30*	0.10	−0.92*	0.64*	0.86*
RS2011DS	−0.66*	0.18	NA	NA	NA

*WS2011RS*: RS experiment of wet season in Nepal; *WS2011NS*: NS experiment of wet season in Nepal; *DS2011RS*: RS experiment of dry season at IRRI; *GY*: Grain yield; *DTF*: Days to 50% flowering; *PHT*: Plant height; *LR*: leaf rolling; *BIO*: Biomass; *HI*: Harvest index.

*: Significant at P ≤ 0.05.

*NR*: LR was not recorded in NS.

*NA*: not available.

### QTL analysis

**Figure 1:**
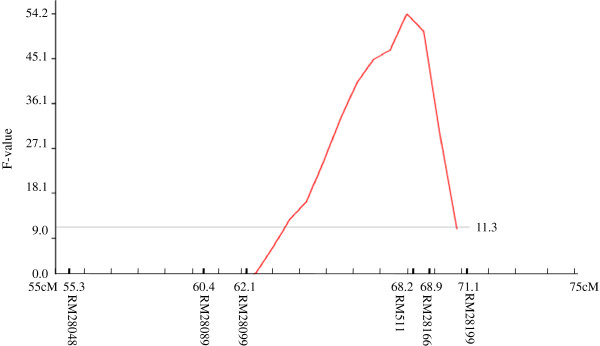
F-statistics for QTL analysis of grain yield under drought stress in the IR74371-46-1-1 × Sabitri population of rice.

QTLs for GY, DTF, PHT, LR, BIO, HI and PAN were identified under RS but none of them were significant in NS situations. A QTL (*qDTY*_*12*.*1*_) on chromosome 12 was identified for GY under RS flanked by markers RM28099 and RM28199 showing a consistent effect in two seasons, DS2011 and WS2011 (Figure 1). At IRRI in DS2011, *qDTY*_*12*.*1*_ explained a phenotypic variance of 3.8% but contributed an additive effect of 22.5% of the trial mean yield, whereas, in WS2011 in Nepal, *qDTY*_*12*.*1*_ explained a phenotypic variance of 38.8% and showed an additive effect of 69.5% of the trial mean yield. In combined analysis over two seasons, this QTL explained a phenotypic variance of 23.8% and additive effect of 45.3% (Table 3). This QTL was significantly associated with PHT in DS2011, and with DTF, LR, BIO, HI and PAN in WS2011 RS experiments. Phenotypic variance explained by this QTL for PHT in DS2011 was 1.1%, contributing an additive effect of 6.3%. Phenotypic variances for DTF, LR, BIO, HI and PAN were 6.5%, 38.6%, 18.0%, 27.4% and 36.0%, respectively. Additive effects contributed by this QTL for DTF, LR, BIO, HI and PAN were 1.5%, -32.6%, 20.9%, 47.1% and 22.3%, respectively. Two other QTLs, one each on chromosomes 2 and 3 (*qDTY*_*2*.*3*_ and *qDTY*_*3*.*2*_), were also found significant for GY under RS in DS2011. *qDTY*_*2*.*3*_ explained a phenotypic variance of 6.5% and an additive effect of -19.7%. *qDTY*_*3*.*2*_ showed phenotypic variance of 7.5% and an additive effect of 20.9% (Table 3).

**Table 3:** QTLs in the IR74371-46-1-1 × Sabitri population associated with GY and related traits under RS

**Trait**	**Chr.**	**QTL name**	**Marker interval**	**CI (cM)**			**Combined**			**DS2011**			**WS2011**		
					**F-value**	**R**^**2**^	**AE%**	**F-value**	**R**^**2**^	**AE%**	**F-value**	**R**^**2**^	**AE%**	
GY	12	*qDTY*_*12*.*1*_	RM28166-RM28199	69.1 – 71.1	48.8	23.8	45.3	8.5	3.8	22.5	104.7	38.8	69.5
	2	*qDTY*_*2*.*3*_	RM3212-RM250	113.1 – 128.5	-	-	-	9.9	6.5	−19.7	-	-	-
	3	*qDTY*_*3*.*2*_	RM22-RM545	5.91 – 9.61	-	-	-	9.9	7.5	20.9	-	-	-
DTF	12	*qDTY*_*12*.*1*_	RM28166-RM28199	69.1 – 71.1	-	-	-	-	-	-	8.4	6.5	1.52
PHT	12	*qDTY*_*12*.*1*_	RM28166-RM28199	69.1 – 71.1	-	-	-	24.7	1.1	6.3	-	-	-
LR	12	*qDTY*_*12*.*1*_	RM28166-RM28199	69.1 – 71.1	51.9	25.1	−22.4	-	-	-	91.3	38.6	−32.6
BIO	12	*qDTY*_*12*.*1*_	RM28166-RM28199	69.1 – 71.1	-	-	-	-	-	-	46.7	18	20.9
HI	12	*qDTY*_*12*.*1*_	RM28166-RM28199	69.1 – 71.1	-	-	-	-	-	-	57.3	27.4	47.1
PAN	12	*qDTY*_*12*.*1*_	RM28166-RM28199	69.1 – 71.1	-	-	-	-	-	-	87.2	36	22.3

*AE*%: Additive effect as a percentage of population mean; *BIO*: Biomass; *CI*: Confidence interval of the QTL (physical distance in Mb); *DTF*: Days to 50% flowering; *GY*: Grain yield; *HI*: Harvest index; *LOD*: Logarithm of odds; *PAN*: Panicle number; *PHT*: Plant height; *LR*: leaf rolling; *R*^*2*^: Phenotypic variance.

### QTL allele analysis

**Figure 2:**
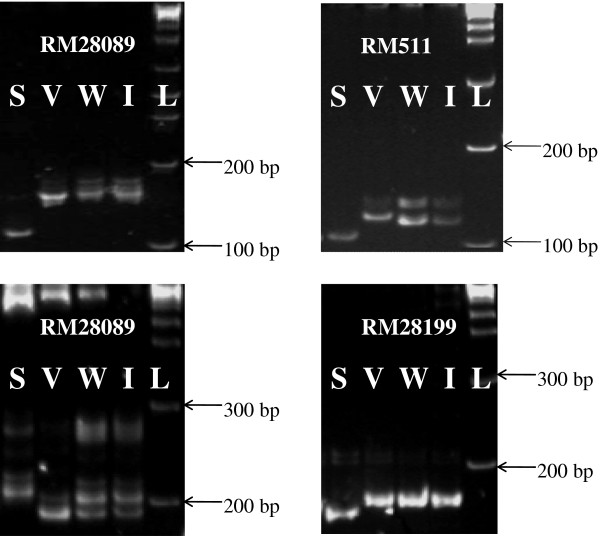
**Allelic pattern of markers RM28089, RM28089, RM511 and RM28199 of qDTY12.1 in Vandana (V), Way Rarem (W), IR74371-46-1-1 (I) and Sabitri (S).** L = 1 Kb Ladder.

*qDTY*_*12*.*1*_ has been identified as a QTL showing an effect under direct-seeded upland conditions [9] in a Vandana/Way Rarem population. Figure 2 presents the results of an allele survey conducted at *qDTY*_*12*.*1*_ locus. It was observed that Way Rarem and IR74371-46-1-1 alleles at RM28089, RM511, RM28166 and RM28199 (markers within the *qDTY*_*12*.*1*_ region) were the same.

## Discussion

Developing rice varieties with high GY under RS is necessary for obtaining sustainable rice yields in drought-prone areas. Popular farmer-accepted varieties could be improved for high GY under RS by following fast-track MAB approaches. Studies have been conducted at IRRI in the past in which large populations developed from crosses between drought-tolerant donors and high-yielding drought-susceptible varieties were used to identify QTLs with a large effect on grain yield under drought [4,9,11,13]. However, large QTL × environment interactions have been reported [23,24] and therefore it is necessary that a QTL for GY under RS show a consistent effect across a wide range of environments for its successful use in MAS.

The identification of a drought GY QTL showing a low QTL × environment interaction effect would be quite useful for MAB purposes. For this, a mapping population needs to be screened at multiple locations. In this study, a population was screened at two locations, IRRI, Philippines, and RARS, Nepal. Dry-season screens at IRRI have been earlier reported to be comparable with wet-season screens in the Indo-Gangetic plains [25]. Phenotypic screening at IRRI, Philippines, and RARS, Nepal, is also comparable as can be seen in this study (Table 1). Previous studies have used this correlation by identifying QTLs through large-scale screening in the dry season at IRRI and validating their effect in the target environment through multi-location testing of a small sub-set of the original mapping population [26]. In this study, we streamlined the QTL identification protocol by combining phenotyping in a target environment (TE) and an effort-saving genotyping method, BSA, for the identification of consistent large-effect QTLs.

Recently, *qDTY*_*1*.*1*_ has been reported to show a large effect against three recipient backgrounds [4]. Earlier, a CT9993-5-10-1-M/IR62266-42-6-2 population was screened at multi-locations and different QTLs were reported to show tolerance by different authors. In a study conducted by Babu et al. [27], QTL *gys1*.*1* affecting grain yield under stress was reported between EM18_10 and L1087 at 113.2-122.2 cM (http://www.gramene.org) on chromosome 1. However, in a study conducted by Kumar et al. [28], the QTL on chromosome 1 was reported between EM11_11 and RG109 with a peak position at 206.6 cM. Babu et al. [27] also reported a QTL (*gys4*.*1*) affecting GY under stress on chromosome 4 between RG939 and RG476 at 103.6-111.7 cM (http://www.gramene.org), which was not reported in the study by Kumar et al. [28]. Despite using the same population, both these studies reported different regions contributing to grain yield under drought. Very few reports of a wide range of consistency exist to date for QTLs for GY under drought. Apart from this report, *qDTY*_*1*.*1*_ reported by Vikram et al. [4] and Ghimire et al. [13] has shown a wide range of effects across donor and recipient backgrounds. However, *qDTY*_*1*.*1*_ also showed an effect only under recipient varieties of the lowland ecosystem.

For a QTL to be widely adopted in a MAS/MAB program, it is necessary that it show a large and consistent effect in different environments, against the genetic background of different recipient drought-susceptible varieties and across ecosystems, upland and lowland. However, in the literature, no such QTL has been reported so far. *qDTY*_*12*.*1*_ has been reported previously in a population derived from the cross of upland cultivars Vandana and Way Rarem, in which it explained a genetic variance of 51% for GY under upland RS [9]. Bernier et al. [9] failed to see any effect of *qDTY*_*12*.*1*_ in lowland RS. Swamy et al. [29] reported the presence of the positive *qDTY*_*12*.*1*_ allele in 85% of the lines from a panel of random drought-tolerant lines.

In this study, high heritability for GY under RS indicates uniformity of drought phenotyping and high stability of the identified QTL. The strong positive correlation of GY with BIO and HI indicates that continued maintenance of biomass production and fertile grain production are the two most important attributes of increased GY under drought.

**Figure 3:**
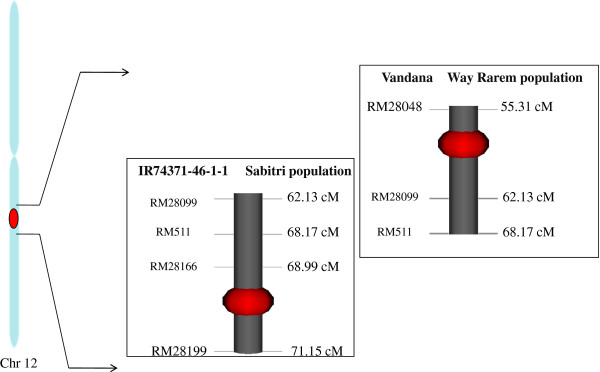
Comparison of the confidence interval of QTLs in a Vandana × Way Rarem population reported by Bernier et al. (2007) and the IR74371-46-1-1 × Sabitri population investigated in this study.

This study identified a large effect of *qDTY*_*12*.*1*_ under lowland RS vis-à-vis upland RS reported earlier by Bernier et al. [9] in a Vandana × Way Rarem population . In this study, *qDTY*_*12*.*1*_ is found to be located on chromosome 12 flanked by markers RM28089 and RM28199 (Figure 1), whereas the QTL interval in an earlier study in a Vandana × Way Rarem population was RM28048 to RM511. The consensus region between the two studies was RM28099-RM511 (Figure 3). This interval could be very important for further genomic studies related to *qDTY*_*12*.*1*_. *qDTY*_*12*.*1*_ showed an additive effect of 45.3% with a phenotypic variance of 23.8% over two years for GY under severe RS conditions (Table 3). It is also important that this QTL has shown a similar high magnitude of effect across the backgrounds of upland-adapted variety Vandana, for which it explained 33% of the phenotypic variance under severe upland drought, and lowland-adapted variety Sabitri, for which it explained 23.8% of the phenotypic variance under severe lowland drought and across environments at IRRI, India [26], and IRRI, Nepal, as found in this study. From this study as well as from an earlier reported study of Bernier et al. [26], it is established beyond doubt that *qDTY*_*12*.*1*_ shows an effect against different recipient genetic backgrounds – upland-adapted Vandana and lowland-adapted Sabitri, different environments – IRRI, India and Nepal, and different ecosystems – upland and lowland. However, phenotypic variance explained by *qDTY*_*12*.*1*_ for GY under RS varied between the DS2011RS experiment at IRRI, Philippines (3.8%), and the WS2011RS experiment at RARS, Nepal (38.8%). This difference could be attributed to QTL × environment and QTL × season interaction effects or higher severity of drought at IRRI than in Nepal. Although there was a difference in phenotypic variance, *qDTY*_*12*.*1*_ showed a consistent and significant effect at both locations. It is evident that the *qDTY*_*1****2***.***1***_ locus is of high importance for MAS, and its pyramiding with other QTLs could have a strong advantage for grain yield under drought.

In contrast to the similar magnitude of effect of this locus seen under lowland RS conditions in this study vis-à-vis the upland ecosystem in an earlier study [9], it is interesting to see that the alleles at RM28166 (the peak marker in this study) are the same for Way Rarem (the donor in the previous study) and IR74371-46-1-1 (the donor in this study). This is obvious because Way Rarem was one of the parents used to develop IR74371-46-1-1. It was also reported recently that the Way Rarem allele at the *qDTY*_*12*.*1*_ locus showed a significant interaction with *qDTY*_*2*.*3*_ (RM573) and *qDTY*_*3*.*2*_ (RM523) regions contributed by the tolerant recipient Vandana [30]. This interaction resulted in a significant effect on GY under lowland RS conditions in a Vandana/Way Rarem population. In this study, QTLs for grain yield under drought were observed in the *qDTY*_*2*.*3*_ and *qDTY*_*3*.*2*_ regions although no interaction was observed.

This study confirmed the effect of *qDTY*_*12*.*1*_ under lowland conditions, which has not been seen before in a Vandana/Way Rarem population. Vandana is a drought-tolerant upland-adapted cultivar and it possesses *qDTY*_*2*.*3*_ and *qDTY*_*3*.*2*_ for grain yield under drought. The appearance of lower drought stress severity under lowland conditions as compared to the severe drought in upland conditions and the tolerance provided to Vandana by *qDTY*_*2*.*3*_ and *qDTY*_*3*.*2*_ in lowland may be the reason for not observing the effect of *qDTY*_*12*.*1*_ contributed by Way Rarem in lowland drought earlier in a Vandana/Way Rarem population. On the other hand, Sabitri is a highly drought-susceptible lowland-adapted cultivar, allowing the effect of the *qDTY*_*12*.*1*_ allele contributed by IR74371-46-1-1 to be seen in lowland even under drought severity lower than that observed in upland. This becomes even more relevant when we see that *qDTY*_*12*.*1*_ as well as *qDTY*_*2*.*3*_ are contributed by IR74371-46-1-1 in this study.

Recently, candidate gene analysis has been carried out in this QTL region and several genes have been reported as putative candidate genes, including a *GRAM*-*domain*-*containing protein*, an *Amydohydrolase*, a *Nodulin MtN3*, a *No Apical Meristem*, a *Cellulose Synthase A* (*CesA10*) and a *cytochrome P450* associated with different processes such as root hair proliferation/elongation, pollen fertility, cell wall permeability and signal transduction [31,32]. Swamy et al. [29] carried out meta-QTL analysis and reported several candidate genes in the same region. *qDTY*_*12*.*1*_ could be efficiently used in marker-assisted breeding for the improvement of both lowland and upland rice varieties for drought stress.

## Conclusions

A major drought grain yield QTL on chromosome 12, *qDTY*_*12*.*1*_, was identified showing a high and consistent effect across two environments – IRRI, Philippines, and RARS, Nepal. The positive allele for *qDTY*_*12*.*1*_ was contributed by the tolerant parent IR74371-46-1-1. Based on a previous study conducted under the upland drought ecosystem in a Vandana/Way Rarem population and this study under the lowland drought ecosystem in an IR74371-46-1-1/Sabitri population, it could be concluded that *qDTY*_*12*.*1*_, *qDTY*_*2*.*3*_ and *qDTY*_*3*.*2*_ are important regions for improving grain yield under drought of susceptible varieties of both lowland and upland ecosystems following MAB.

## Abbreviations

BIL: Backcross inbred line; BIO: Biomass; BSA: Bulked segregant analysis; CTAB: Cetyl trimethyl ammonium bromide; DAT: Days after transplanting; DNA: Deoxyribonucleic acid; DS: Dry season; DTF: Days to 50% flowering; GY: Grain yield; HI: Harvest index; IRRI: International Rice Research Institute; LOD: Logarithm of odds; LR: Leaf rolling; MAB: Marker-assisted breeding; NPK: Nitrogen, phosphorus, and potassium; NS: Non-stress; PAN: Panicle number; PCR: Polymerase chain reaction; PAGE: Polyacrylamide gel electrophoresis; PHT: Plant height; QTL: Quantitative trait loci; R2: Phenotypic variance; RARS: Regional Agricultural Research Station, Nepalgunj, Nepal; RS: Reproductive-stage drought stress; SNP: Single nucleotide polymorphism; SSR: Simple sequence repeats; WS: Wet season.

## Competing interests

Authors declare that there were no competing interests.

## Authors’ contributions

KKM was involved in the analysis, interpretation of the data and drafting the; PV was associated with the analysis and interpretation of the data and the revision of the manuscript; RBY was involved in the conception of the experiment and drafting the article; BPMS, SD, MTSC, PM and SM helped in the analysis and interpretation of the data and revised the manuscript; AK was involved in the conception and design of the experiment and the critical revision of the manuscript. All authors approved the final version of the manuscript.

## Supplementary Material

Additional file 1 Table presents the details of primers used in this study. Click here for file

Additional file 2 Water table and rainfall data of DS2011 stress experiment at IRRI, Philippines. Click here for file

Additional file 3 Water table and rainfall data of WS2011 stress experiment at RARS, Nepalgunj, Nepal. Click here for file

## References

[B1] Pandey S Bhandari H Hardy B Economic Costs of Drought and Rice Farmers’ Coping Mechanisms. A Cross-Country Comparative Analysis 2007 Los Baños, Philippines/Singapore: International Rice Research Institute/World Scientific Publishing 1 9

[B2] Huke RE Huke EH Rice area by type of culture: South, Southeast, and East Asia 1997 Los Baños, Philippines: IRRI

[B3] Evenson RE Gollin D Assessing the impact of the green revolution, 1960 to 2000 Science 2003 300 758 762 10.1126/science.1078710 12730592

[B4] Vikram P Swamy BPM Dixit S Ahmed HU Sta Cruz MT Singh AK Kumar A *qDTY*_*1*.*1*_, major QTL for rice GY under reproductive-stage drought stress with a consistent effect in multiple elite genetic backgrounds BMC Genet 2011 12 89 22008150 10.1186/1471-2156-12-89 PMC3234187

[B5] Fukai S Cooper M Development of drought resistant cultivars using physio-morphological traits in rice Field Crops Res 1995 40 67 86 10.1016/0378-4290(94)00096-U

[B6] Kumar A Bernier J Verulkar S Lafitte HR Atlin GN Breeding for drought tolerance: direct selection for yield, response to selection and use of drought-tolerant donors in upland and lowland-adapted populations Field Crops Res 2008 107 221 231 10.1016/j.fcr.2008.02.007

[B7] Venuprasad R Lafitte HR Atlin GN Response to direct selection for grain yield under drought stress in rice Crop Sci 2007 47 285 293 10.2135/cropsci2006.03.0181

[B8] Kumar A Verulkar SB Mandal NP Variar M Shukla VD Dwivedi JL Singh BN Singh ON Swain P Mall AK Robin S Chandrababu R Jain A Haefele SM Piepho HP Raman A High- yielding, drought-tolerant, stable rice genotypes for the shallow rainfed lowland drought prone ecosystem Field Crops Res 2012 133 37 47

[B9] Bernier J Kumar A Venuprasad R Spaner D Atlin GN A large-effect QTL for GY under reproductive-stage drought stress in upland rice Crop Sci 2007 47 507 516 10.2135/cropsci2006.07.0495

[B10] Swamy BPM Kumar A Teang S Sustainable rice yield in water short drought prone environments: conventional and molecular approaches Irrigation systems and practices in challenging environments 2011 Janeza Trdine 9, 51000 Rijeka, Croatia: InTech 149 168

[B11] Venuprasad R Dalid CO Del Valle M Zhao D Espiritu M Sta Cruz MT Amante M Kumar A Atlin GN Identification and characterization of large-effect quantitative trait loci for GY under lowland drought stress in rice using bulk-segregant analysis Theor Appl Genet 2009 120 177 190 10.1007/s00122-009-1168-1 19841886

[B12] Salekdeh GH Siopongco J Wade LJ Ghareyazie B Bennett J A proteomic approach to analyzing drought and salt responsiveness in rice Field Crops Res 2002 76 199 219 10.1016/S0378-4290(02)00040-0

[B13] Ghimire KH Quiatchon L Vikram P Swamy BPM Dixit S Ahmed HU Hernandez JE Borromeo TH Kumar A Identification and mapping of QTL (*qDTY*_*1*.*1*_) with a consistent effect on GY under RS Field Crops Res 2012 131 88 96 10.1016/j.fcr.2012.02.028

[B14] Michelmoore RW Paran I Kesseli RV Identification of markers linked to disease resistance genes by bulked segregant analysis: a rapid method to detect markers in specific genomic regions by using segregating populations Proc Natl Acad Sci USA 1991 88 9828 9832 10.1073/pnas.88.21.9828 1682921 PMC52814

[B15] Murray MG Thompson WF Rapid isolation of high molecular weight plant DNA Nucl Acids Res 1980 8 4321 4325 10.1093/nar/8.19.4321 7433111 PMC324241

[B16] Sambrook J Fritsch EF Maniatis T Molecular cloning: a laboratory manual 1989 2 New York: Cold Spring Harbor

[B17] Temnykh S Declerck G Lukashova A Lipovich L Cartinhour S McCouch S Computational and experimental analysis of microsatellites in rice (*Oryza sativa* L.): frequency, length variation, transposon associations, and genetic marker potential Genome Res 2001 11 1441 1452 10.1101/gr.184001 11483586 PMC311097

[B18] McCouch SR Teytelman L Xu Y Lobos KB Clare K Walton M Fu B Maghirang R Li Z Xing Y Zhang Q Kono I Yano M Fjellstrom R DeClerck G Schneider D Cartinhour S Ware D Stein L Development and mapping of 2240 new SSR markers for rice (*Oryza sativa* L.) DNA Res 2002 9 199 207 10.1093/dnares/9.6.199 12597276

[B19] IRGSP The map-based sequence of the rice genome Nature 2005 436 793 800 10.1038/nature03895 16100779

[B20] Vikram P Swamy BPM Dixit S Ahmed HU Sta Cruz MT Singh AK Ye G Kumar A Bulk segregant analysis: An effective approach for mapping drought grain yield QTLs in rice Field Crops Res 2012 134 185 192

[B21] Yang J Zhu J Williams RW Mapping the genetic architecture of complex traits in experimental populations Bioinformatics 2007 23 527 536 10.1093/bioinformatics/btm007 17459962

[B22] Joehanes R Nelson JC QGene 4.0, an extensible java QTL-analysis platform Bioinformatics 2008 24 2788 2789 10.1093/bioinformatics/btn523 18940826

[B23] Price AH Cairns JE Horton P Jones RGW Griffiths H Linking drought-resistance mechanisms to drought avoidance in upland rice during a QTL approach: progress and new opportunities to integrate stomatal and mesophyll responses J Exp Bot 2002 53 989 1004 10.1093/jexbot/53.371.989 11971911

[B24] Xing YZ Tan YF Hua JP Sun XL Xu CG Zhang Q Characterization of the main effects, epistatic effects and their environmental interactions of QTLs on the genetic basis of yield traits in rice Theor Appl Genet 2002 105 248 257 10.1007/s00122-002-0952-y 12582526

[B25] Verulkar SB Mandal NP Dwivedi JL Singh BN Sinha PK Mahato RN Swain P Dongre P Payasi D Singh ON Bose LK Robin S Babu RC Senthil S Jain A Shashidhar HE Hittalmani S Vera Cruz C Paris T Hijmans R Raman A Haefele S Serraj R Atlin G Kumar A Breeding resilient and productive rice genotypes adapted to drought-prone rainfed ecosystems of India Field Crops Res 2010 117 197 208 10.1016/j.fcr.2010.03.005

[B26] Bernier J Kumar A Spaner D Verulkar S Mandal NP Sinha PK Peeraju P Dongre PR Mahto RN Atlin GN Characterization of the effect of rice drought tolerance qtl12.1 over a range of environments in the Philippines and eastern India Euphytica 2009 166 207 217 10.1007/s10681-008-9826-y

[B27] Babu RC Nguyen BD Chamarerk V Shanmugasundaram P Chezhian P Jeyaprakash P Ganesh SK Palchamy A Sadasivam S Sarkarung S Wade LJ Nguyen HT Genetic analysis of drought resistance in rice by molecular markers: association between secondary traits and field performance Crop Sci 2003 43 1457 1469 10.2135/cropsci2003.1457

[B28] Kumar R Venuprasad R Atlin GN Genetic analysis of rainfed lowland rice drought tolerance under naturally-occurring stress in eastern India: heritability and QTL effects Field Crops Res 2007 103 42 52 10.1016/j.fcr.2007.04.013

[B29] Swamy BPM Vikram P Dixit S Ahmed HU Kumar A Meta-analysis of GY QTL identified during agricultural drought in grasses showed consensus BMC Genomics 2011 12 319 10.1186/1471-2164-12-319 21679437 PMC3155843

[B30] Dixit S Swamy BPM Vikram P Bernier J Sta Cruz MT Amante M Atri D Kumar A Increased drought tolerance and wider adaptability of *qDTY*_*12*.*1*_ conferred by its interaction with *qDTY*_*2*.*3*_ and *qDTY*_*3*.*2*_ Mol Breed 2012 30 1767 1779 10.1007/s11032-012-9760-5

[B31] Kohli A Narciso J Oane R Popluechai S Kumar A Identification of major candidate genes in a large effect QTL for rice yield under drought stress Paper presented at International Rice Congress, Hanoi, Vietnam, 9–11 November, 2010

[B32] Biswal AK Oane R Raorane M Narciso J Blesilda AE Kumar A Kohli A Identification of candidate genes in the large effect QTL DTY12.1 for yield under stress Presented at FCSSP Conference, held in Puerto Princesa City, Palawan, Philippines, April 16–21, 2012

