# Supplementary material for: qDTY12.1: a locus with a consistent effect on grain yield under drought in rice
Source: BMC Genet. 2013 Feb 26;14:12. doi: 10.1186/1471-2156-14-12 (PMC3616849; doi:10.1186/1471-2156-14-12)
Supplement: Additional file 1 — Table presents the details of primers used in this study. [file 1471-2156-14-12-S1.docx]

Supplementary table 1: Details of the primers used for QTL analysis

| Primer name | Chromosome | Physical position (Mb) |  | Primer sequences |
| --- | --- | --- | --- | --- |
| RM3212 | 2 | 28.8 | F | AGACGACAAACACCTGCCTC |
|  |  |  | R | CAAACACAAACGCAGCCTC |
| RM250 | 2 | 32.7 | F | GGTTCAAACCAAGCTGATCA |
|  |  |  | R | GATGAAGGCCTTCCACGCAG |
| RM208 | 2 | 35.2 | F | TCTGCAAGCCTTGTCTGATG |
|  |  |  | R | TAAGTCGATCATTGTGTGGACC |
| RM22 | 3 | 1.5 | F | GGTTTGGGAGCCCATAATCT |
|  |  |  | R | CTGGGCTTCTTTCACTCGTC |
| RM545 | 3 | 4.9 | F | CAATGGCAGAGACCCAAAAG |
|  |  |  | R | CTGGCATGTAACGACAGTGG |
| RM28048 | 12 | 14.1 | F | TTCAGCCGATCCATTCAATTCC |
|  |  |  | R | GCTATTGGCCGGAAAGTAGTTAGC |
| RM28089 | 12 | 15.4 | F | GGGAGGACACCTGTGTAAGTAGG |
|  |  |  | R | GGTTCAAATGAGACCCAATTCC |
| RM28099 | 12 | 15.8 | F | TGTGCGGATGCGGGTAAGTCC |
|  |  |  | R | CCACCTGTCAACCACCGAAACC |
| RM511 | 12 | 17.4 | F | CTTCGATCCGGTGACGAC |
|  |  |  | R | AACGAAAGCGAAGCTGTCTC |
| RM28166 | 12 | 17.6 | F | TGCTTGCAAACATTGCTTCTGG |
|  |  |  | R | ACTGATGTACTGAACACGGGAAGG |
| RM28199 | 12 | 18.2 | F | CGGCTTAGGGAGCGTCTGTAGG |
|  |  |  | R | GCATGCTAGTATGGCCACCATATTCC |
