# Supplementary figures and images for: qDTY12.1: a locus with a consistent effect on grain yield under drought in rice
Source: BMC Genet. 2013 Feb 26;14:12. doi: 10.1186/1471-2156-14-12 (PMC3616849; doi:10.1186/1471-2156-14-12)

## Slide 1
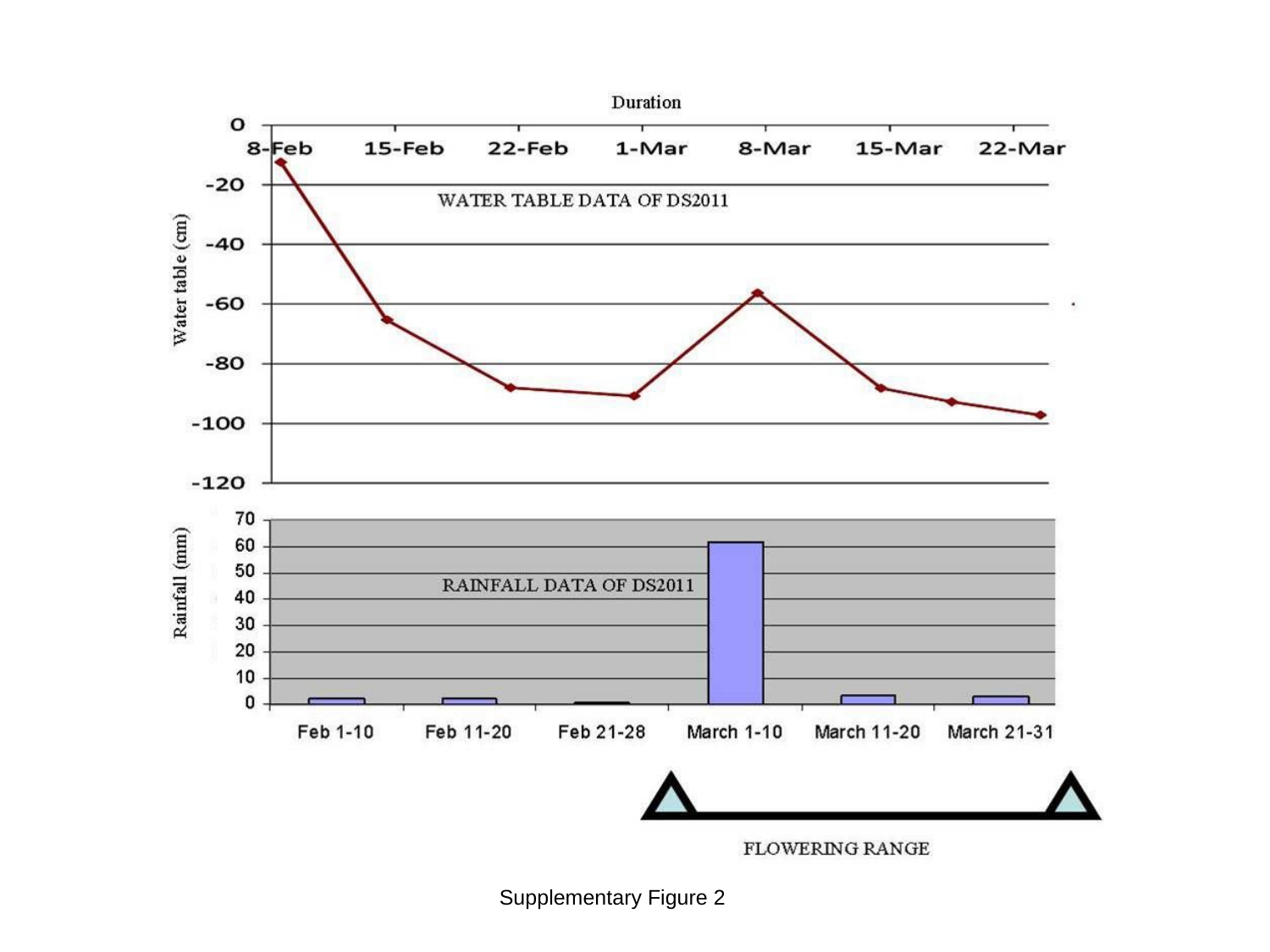

WATER TABLE DATA OF DS2011
Supplementary Figure 2

Supplement: Additional file 2 — Water table and rainfall data of DS2011 stress experiment at IRRI, Philippines. [file 1471-2156-14-12-S2.pptx]

## Slide 1
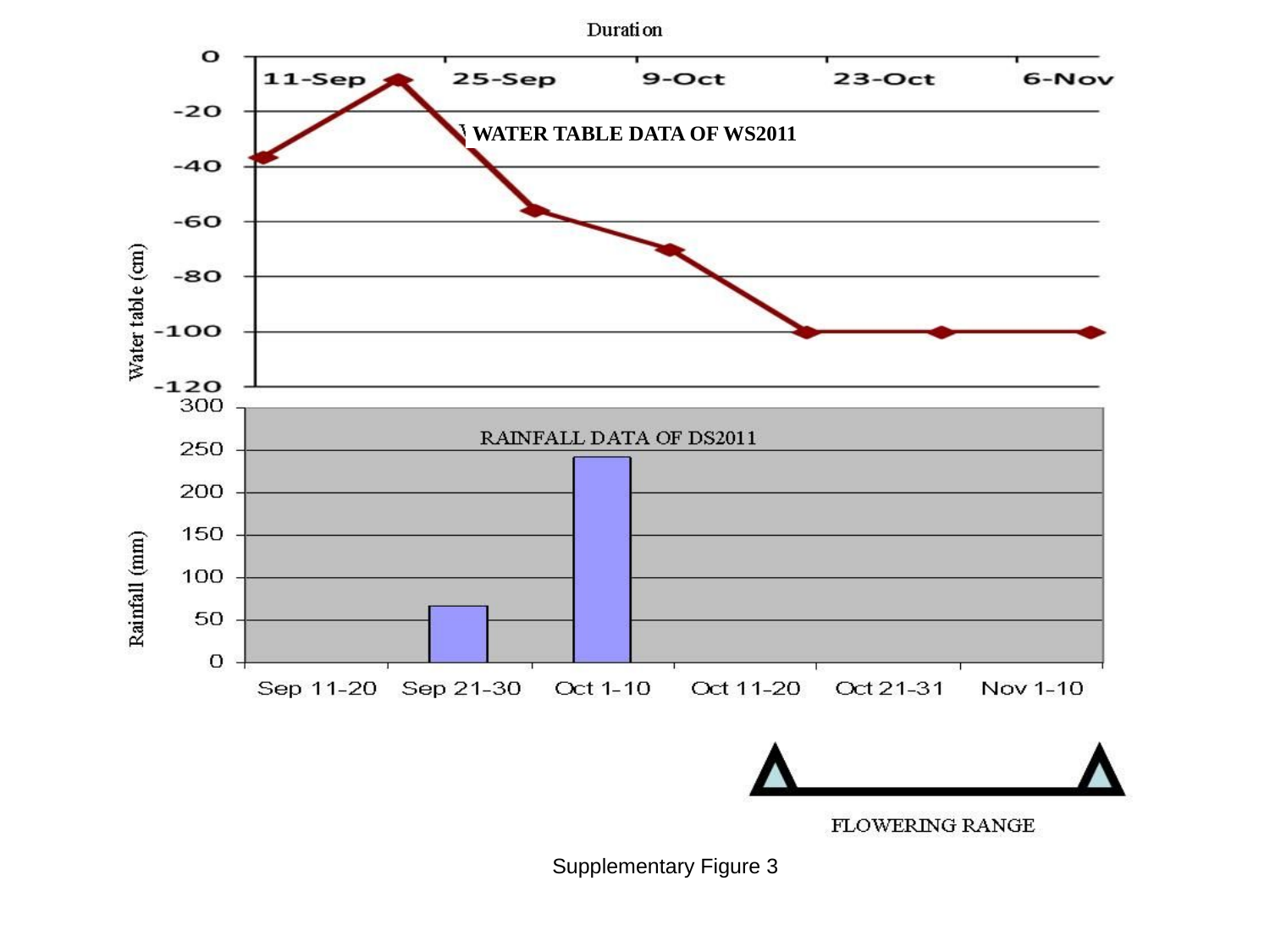

WATER TABLE DATA OF WS2011
Supplementary Figure 3

Supplement: Additional file 3 — Water table and rainfall data of WS2011 stress experiment at RARS, Nepalgunj, Nepal. [file 1471-2156-14-12-S3.pptx]
